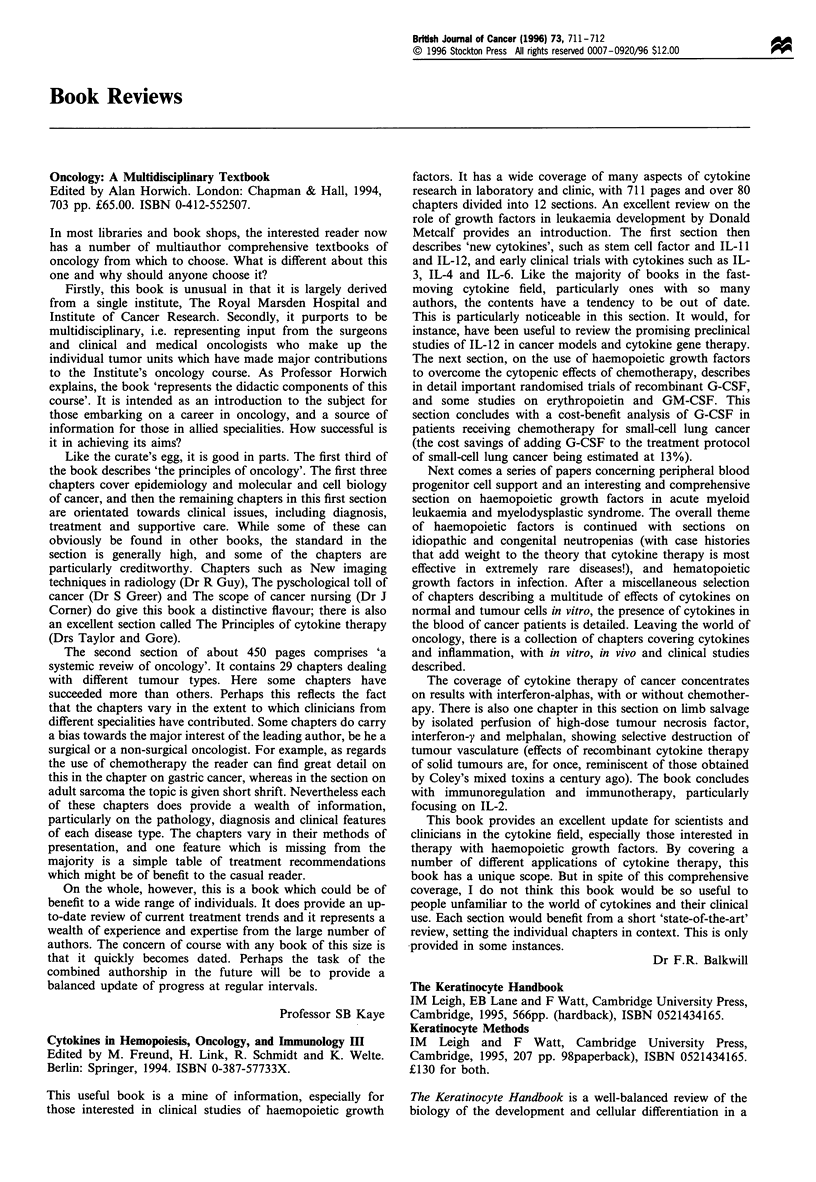# Oncology: A Multidisciplinary Textbook

**Published:** 1996-03

**Authors:** SB Kaye


British Journal of Cancer (1996) 73, 711-712
© 1996 Stockton Press All rights reserved 0007-0920/96 $12.00

### Book Reviews

**Oncology: A Multidisciplinary Textbook**
Edited by Alan Horwich. London: Chapman & Hall, 1994, 703 pp. £65.00. ISBN 0-412-552507.

In most libraries and book shops, the interested reader now has a number of multiauthor comprehensive textbooks of oncology from which to choose. What is different about this one and why should anyone choose it?

Firstly, this book is unusual in that it is largely derived from a single institute, The Royal Marsden Hospital and Institute of Cancer Research. Secondly, it purports to be multidisciplinary, i.e. representing input from the surgeons and clinical and medical oncologists who make up the individual tumor units which have made major contributions to the Institute's oncology course. As Professor Horwich explains, the book 'represents the didactic components of this course'. It is intended as an introduction to the subject for those embarking on a career in oncology, and a source of information for those in allied specialities. How successful is it in achieving its aims?

Like the curate's egg, it is good in parts. The first third of the book describes 'the principles of oncology'. The first three chapters cover epidemiology and molecular and cell biology of cancer, and then the remaining chapters in this first section are orientated towards clinical issues, including diagnosis, treatment and supportive care. While some of these can obviously be found in other books, the standard in the section is generally high, and some of the chapters are particularly creditworthy. Chapters such as New imaging techniques in radiology (Dr R Guy), The pyschological toll of cancer (Dr S Greer) and The scope of cancer nursing (Dr J Corner) do give this book a distinctive flavour; there is also an excellent section called The Principles of cytokine therapy (Drs Taylor and Gore).

The second section of about 450 pages comprises 'a systemic reveiw of oncology'. It contains 29 chapters dealing with different tumour types. Here some chapters have succeeded more than others. Perhaps this reflects the fact that the chapters vary in the extent to which clinicians from different specialities have contributed. Some chapters do carry a bias towards the major interest of the leading author, be he a surgical or a non-surgical oncologist. For example, as regards the use of chemotherapy the reader can find great detail on this in the chapter on gastric cancer, whereas in the section on adult sarcoma the topic is given short shrift. Nevertheless each of these chapters does provide a wealth of information, particularly on the pathology, diagnosis and clinical features of each disease type. The chapters vary in their methods of presentation, and one feature which is missing from the majority is a simple table of treatment recommendations which might be of benefit to the casual reader.

On the whole, however, this is a book which could be of benefit to a wide range of individuals. It does provide an up-to-date review of current treatment trends and it represents a wealth of experience and expertise from the large number of authors. The concern of course with any book of this size is that it quickly becomes dated. Perhaps the task of the combined authorship in the future will be to provide a balanced update of progress at regular intervals.

Professor SB Kaye